# Prostate stem cell antigen (*PSCA*) and risk of bladder cancer: linking genotypes to functional mechanisms

**DOI:** 10.1186/gb-2011-12-s1-p15

**Published:** 2011-09-19

**Authors:** Adam Mumy, Indu Kohaar, Patricia Porter-Gill, Wei Tang, Yi-Ping Fu, Ludmila Prokunina-Olsson

**Affiliations:** 1 Laboratory of Translational Genomics, Division of Cancer Epidemiology and Genetics, National Cancer Institute, National Institutes of Health, Bethesda, MD 20892, USA

## Background

Genome-wide association studies (GWAS) have identified a single nucleotide polymorphism, rs2294008 C/T, within the prostate stem cell antigen (*PSCA*) gene as a risk variant for bladder cancer [1]. PSCA is a glycosyl phosphatidylinositol (GPI)-anchored cell surface protein from the Ly-6/Thy-1 family of cell surface antigens. PSCA overexpression has been reported in bladder, prostate and pancreatic tumors. The risk allele (T) of rs2294008 creates a novel translation start site and extends the PSCA leader peptide sequence by 11 amino acids.

## Methods

The mRNA expression in 42 bladder tumor samples and 39 adjacent normal bladder tissue samples (24 matched normal-tumor pairs) was explored using genome-wide RNA sequencing and targeted *PSCA* mRNA expression assays. For allelic expression imbalance studies, genotyping of rs2294008 both in DNA and cDNA samples was performed using an allelic discrimination genotyping assay. Alternative allele-specific splicing forms of *PSCA* were cloned and transfected into several human cancer cell lines. The endogenous expression of PSCA protein and the expression pattern of the recombinant PSCA allelic isoforms in different cancer cell lines were studied by western blotting, confocal microscopy and fluorescence-activated cell-sorting analysis. PSCA protein expression in normal and tumor bladder tissue samples was examined in relation to rs2294008 genotypes by using immunohistochemistry.

## Results

*PSCA* mRNA was expressed at a 5.7-fold higher level in tumors than in matching normal bladder tissue samples (*P* = 0.0060). There was a strong allelic expression imbalance in tumor samples (*P* = 0.0020), based on 20 normal and 13 tumor samples that were heterozygous for rs2294008. *PSCA* mRNA expression was associated with the genotype of rs2294008 both in normal and tumor bladder tissue samples. Our preliminary data on the expression of recombinant allele-specific PSCA protein isoforms in transfected cells show a possible difference in the distribution of the cytoplasmic and membrane expression of these isoforms.

## Conclusions

Our results suggest that the extension of the PSCA leader peptide by 11 amino acids, introduced by the risk allele (T) of rs2294008, may affect subcellular protein localization and the availability of functional GPI-anchored PSCA on the cell surface. These results may have clinical implications because antibodies that target cell-surface-expressed PSCA are in clinical trials for pancreatic and prostate cancer.
